# Optical Sensing to Determine Tomato Plant Spacing for Precise Agrochemical Application: Two Scenarios

**DOI:** 10.3390/s17051096

**Published:** 2017-05-11

**Authors:** Jorge Martínez-Guanter, Miguel Garrido-Izard, Constantino Valero, David C. Slaughter, Manuel Pérez-Ruiz

**Affiliations:** 1 Aerospace Engineering and Fluid Mechanics Department, University of Seville, 41013 Seville, Spain; martinezj@us.es; 2 Laboratorio de Propiedades Físicas (LPF_TAGRALIA), Universidad Politécnica de Madrid (UPM), 28040 Madrid, Spain; miguel.garrido.izard@upm.es (M.G.-I.); constantino.valero@upm.es (C.V.); 3 Department of Biological and Agricultural Engineering, University of California, Davis, CA 95616, USA; dcslaughter@ucdavis.edu

**Keywords:** LiDAR, light-beam, plant localization, Kinect

## Abstract

The feasibility of automated individual crop plant care in vegetable crop fields has increased, resulting in improved efficiency and economic benefits. A systems-based approach is a key feature in the engineering design of mechanization that incorporates precision sensing techniques. The objective of this study was to design new sensing capabilities to measure crop plant spacing under different test conditions (California, USA and Andalucía, Spain). For this study, three different types of optical sensors were used: an optical light-beam sensor (880 nm), a Light Detection and Ranging (LiDAR) sensor (905 nm), and an RGB camera. Field trials were conducted on newly transplanted tomato plants, using an encoder as a local reference system. Test results achieved a 98% accuracy in detection using light-beam sensors while a 96% accuracy on plant detections was achieved in the best of replications using LiDAR. These results can contribute to the decision-making regarding the use of these sensors by machinery manufacturers. This could lead to an advance in the physical or chemical weed control on row crops, allowing significant reductions or even elimination of hand-weeding tasks.

## 1. Introduction

Precision agriculture requires accurate plant or seed distribution across a field. This distribution is to be optimized according to the size and shape of the area in which nutrients and light are provided to plant to obtain the maximum possible yield. These factors are controlled by the spacing between crop rows and the spacing of plants/seeds in a row [1]. For many crops, row spacing is determined as much by the physical characteristics of agricultural machinery used to work in the field as by the specific biological spacing requirements of the crop [2]. According to the crop and machinery used, the accuracy of planting by the precision transplanter/seeder to the desired square grid pattern must be adequate for the operation of agricultural machinery in both longitudinal and transverse crop directions.

The current designs of vegetable crop transplanters and seeders utilize several uncoordinated planting modules mounted to a common transport frame. These systems use sub-optimal open-loop methods that neglect the dynamic and kinematic effects of the mobile transport frame and of plant motion relative to the frame and the soil. The current designs also neglect to employ complete mechanical control of the transplant during the entire planting process, producing an error in the final planting position, due to the increased uncertainty of plant location as a result of natural variations in plant size, plant mass, soil traction and soil compaction [3].

Accurately locating the crop plant, in addition to allowing automatic control of weeds, allows individualized treatment of each plant (e.g., spraying, nutrients). Seeking to ensure minimum physical interaction with plants (i.e., non-contact), different remote sensing techniques have been used for the precise localization of plants in fields. For these localization methods, some authors have decided to address automatic weed control by localizing crop plants with centimetre accuracy during seed drilling [4] or transplanting [5,6] using a global positioning system in real time (RTK-GNSS). These studies, conducted at UC Davis, have shown differences between RTK-GNSS-based expected seed location versus actual plant position. The position uncertainly ranged from 3.0 to 3.8 cm for seeds, and tomato transplants, the mean system RMS was 2.67 cm in the along-track direction. Nakarmi and Tang used an image acquisition platform after planting to estimate the inter-plant distance along the crop rows [7]. This system could measure inter-plant distance with a minimum error of ±30 cm and a maximum error of ±60 cm.

Today, one of the biggest challenges to agricultural row crop production in industrialized countries is non-chemical control of intra-row (within the crop row) weed plants. Systems such as those developed by Pérez-Ruiz et al. [8] or the commercial platforms based on computer-controlled hoes developed by Dedousis et al. [9] are relevant examples of innovative mechanical weeding systems. However, the current effectiveness of mechanical weed removal is constrained by plant spacing, the proximity of the weeds to the plant, the plant height and the operation timing. Other methods for non-chemical weed control, such as the robotic platform developed by Blasco et al. [10] (capable of killing weeds using a 15-kV electrical discharge), the laser weeding system developed by Shah et al. [11] or the cross-flaming weed control machine designed for the RHEA project by Frasconi et al. [12], demonstrate that research to create a robust and efficient system is ongoing. A common feature of all these technological developments is the need for accurate measurement of the distance between plants.

Spatial distribution and plant spacing are considered key parameters for characterizing a crop. The current trend is towards the use of optical sensors or image-based devices for measurements, despite the possible limitations of such systems under uncontrolled conditions such as those in agricultural fields. These image-based tools aim to determine and accurately correlate several quantitative aspects of crops to enable plant phenotypes to be estimated [13,14].

Dworak et al. [15] categorized research studying inter-plant location measurements into two types: airborne and ground-based. Research on plant location and weed detection using airborne sensors has increased due to the increasing potential of unmanned aerial systems in agriculture, which have been used in multiple applications in recent years [16]. For ground-based research, one of the most widely accepted techniques for plant location and classification is the use of Light Detection and Ranging (LiDAR) sensors [17]. These sensors provide distance measurements along a line scan at a very fast scanning rate and have been widely used for various applications in agriculture, including 3D tree representation for precise chemical applications [18,19] or in-field plant location [20]. This research continues the approach developed in [21], in which a combination of LiDAR + IR sensors mounted on a mobile platform was used for the detection and classification of tree stems in nurseries.

Based on the premise that accurate localization of the plant is key for precision chemical or physical removal of weeds, we propose in this paper a new methodology to precisely estimate tomato plant spacing. In this work, non-invasive methods using optical sensors such as LiDAR, infrared (IR) light-beam sensors and RGB-D cameras have been employed. For this purpose, a platform was developed on which different sensor configurations have been tested in two scenarios: North America (UC Davis, CA, USA) and Europe (University of Seville, Andalucia, Spain). The specific objectives, given this approach, were the following:

- To design and evaluate the performance of multi-sensor platforms attached to a tractor (a UC Davis platform mounted on the rear of the tractor and a University of Seville platform mounted on the front of the tractor).
- To refine the data-processing algorithm to select the most reliable sensor for the detection and localization of each tomato plant.

## 2. Materials and Methods

To develop a new sensor platform to measure the space between plants in the same crop row accurately, laboratory and field tests were conducted in Andalucia (Spain) and in California (USA). This allowed researchers to obtain more data under different field conditions and to implement the system improvements required, considering the plant spacing objective. These tests are described below, characterizing the sensors used and the parameters measured.

### 2.1. Plant Location Sensors

#### 2.1.1. Light-Beam Sensor Specifications

**Figure 1:**
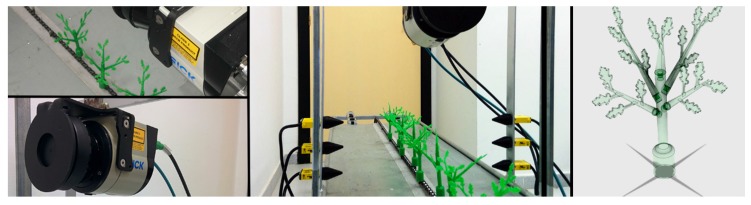
Details of the sensors on the laboratory platform (vertical LiDAR and Light-beam sensors) for the detection and structure of the modular 3D plant.

**Figure 2:**
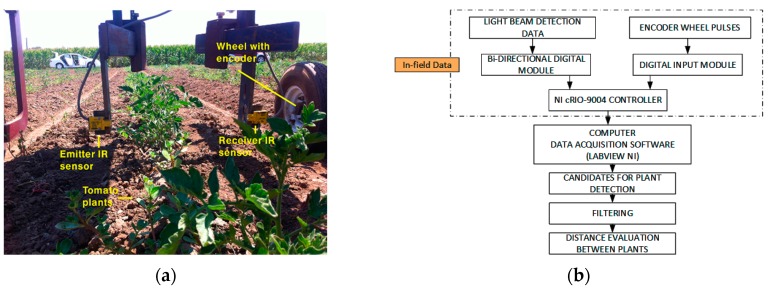
(**a**) Light-beam sensors mounted on the experimental platform designed for field trials at UC Davis, California; (**b**) Progressive monitoring flowchart using light-beam sensors.

IR light-beam sensors (Banner SM31 EL/RL, Banner Engineering Co., Minneapolis, MN, USA) were used in two configurations: first as a light curtain (with three pairs of sensors set vertically, Figure 1 central and Figure 4) and later a simpler setup, using only one pair of sensors (Figure 2), which simplifies the system while still allowing the objective (plant spacing measurement) to be attained. In the light curtain, light-beam sensors were placed transversely in the middle of the platform to detect and discriminate the plant stem in a cross configuration to prevent crossing signals between adjacent sensors. Due to the short range and focus required in laboratory tests, it was necessary to reduce the field of view and the strength of the light signal by masking the emitter and receiver lens with a 3D-printed conical element. In laboratory tests, the height of the first emitter and receiver pair above the platform was 4 cm, and the height of 3D plants (artificial plants were used in laboratory tests; see Section 2.2) was 13 cm. In the field tests, the sensor was placed 12 cm from the soil (the average height measured manually for real plants in outdoor tests was 19.5 cm) to avoid obstacles in the field (e.g., dirt clods, slight surface undulations). In both cases, the receiver was set to obtain a TTL output pulse each time the IR light-beam was blocked by any part of the plant. The signals generated by the sensors were collected and time-stamped by a microcontroller in real time and stored for off-line analysis. Technical features of the IR light-beam sensors are presented in Table 1.

#### 2.1.2. Laser Scanner

A LMS 111 LiDAR laser scanner (SICK AG, Waldkirch, Germany), was used in the laboratory and field testing platforms to generate a high-density point cloud on which to perform the localization measurements. Its main characteristics are summarized in Table 2. The basic operating principle of the LiDAR sensor is the projection of an optical signal onto the surface of an object at a certain angle and range. Processing the corresponding reflected signal allows the sensor to determine the distance to the plant. The LiDAR sensor was interfaced with a computer through an RJ 45 Ethernet port for data recording. Data resolution was greatly affected by the speed of the platform’s movement; thus, maintenance of a constant speed was of key importance for accurate measurements. During data acquisition, two digital filters were activated for optimizing the measured distance values: a fog filter (becoming less sensitive in the near range (up to approximately 4 m)); and an N-pulse-to-1-pulse filter, which filters out the first reflected pulse in case that two pulses are reflected by two objects during a measurement [22]. Different LiDAR scan orientations were evaluated: scanning vertically with the sensor looking downwards (Figure 1), scanning with a 45° inclination (push-broom) and a lateral-scanning orientation (side-view).

#### 2.1.3. RGB-D Camera

A Kinect V2 commercial sensor (Microsoft, Redmond, WA, USA), originally designed for indoor video games, was mounted sideways on the research platform during field trials. This sensor captured RGB, NIR and depth images (based on time-of-flight) of tomato plants, although for further analysis, only RGB images were used for the validation of stick/tomato locations obtained from the LiDAR scans, as detailed in Section 2.4.3. Kinect RGB-captured images have a resolution of 1920 × 1080 pixels and a field of view (FOV) of 84.1 × 53.8°, resulting in an average of approximately 22 × 20 pixels per degree. NIR images and depth camera have a resolution of 512 × 424 pixels, with an FOV of 70 × 60° and a depth-sensing maximum distance of 4.5–5 m. Although systems such as the Kinect sensor were primarily designed for use under controlled light conditions, the second version of this sensor has higher RGB resolution (640 × 480 in v1) and its infrared sensing capabilities were also improved, enabling a more lighting-independent view and supporting its use outdoors under high-illumination conditions. Despite this improvement, we observed that the quality of the RGB images were somewhat affected by luminosity and direct incident light, and therefore, the image must be post-processed to obtain usable results. The images taken by the Kinect sensor were simultaneously acquired and synchronized with the LiDAR scans and the encoder pulses. Because the LabVIEW software (National Instruments, Austin, TX, USA) used for obtaining the scan data was developed to collect three items (the scans themselves, the encoder pulses and the timestamp), a specific Kinect recording software had to be developed to embed the timestamp value in the image data. With the same timestamp for the LiDAR and the image, the data could be matched and the images used to provide information about the forward movement of the platform. 

### 2.2. Lab Platform Design and Tests

To maximize the accuracy of the distance measurements obtained by the sensors, an experimental platform was designed to avoid the seasonal limitations of testing outdoors. Instead of working in a laboratory with real plants, the team designed and created model plants (see Figure 1) using a 3D printer (Prusa I3, BQ, Madrid, Spain). These plants were mounted on a conveyor chain at a predetermined distance. This conveyor chain system, similar to that of a bicycle, was driven by a small electric motor able to move the belt at a constant speed of 1.35 km·h^−1^. For the odometry system, the shaft of an incremental optical encoder (63R256, Grayhill Inc., Chicago, IL, USA) was mounted so that it was attached directly to the gear shaft and used to measure the distance travelled, thus serving as a localization reference system. Each channel in this encoder generates 256 pulses per revolution, providing a 3-mm resolution in the direction of travel. The data generated by the light-beam sensors and the cumulative odometer pulse count were collected using a low-cost open-hardware Arduino Leonardo microcontroller (Arduino Project, Ivrea, Italy) programmed in a simple integrated development environment (IDE). This device enabled recording of data that were stored in a text file for further computer analysis. Several repetitions of the tests were made on the platform to optimize the functions of both light-beam and LiDAR sensors. From the three possible LiDAR orientations, lateral scanning was selected for the field trials because it provided the best information on the structure of the plant, as concluded in [17]. In lab tests, two arrangements of light-beam sensors were assessed: one in a light curtain assembly with three sensor pairs at different heights and another using only one emitter-receiver pair.

### 2.3. Field Tests

The initial tests, performed in Davis, CA (USA), were used to assess the setup of the light-beam sensor system and detected only the stem of the plants rather than locating it within a local reference system. Once the tomato plants were placed in the field, tests were conducted at the Western Center for Agriculture Equipment (WCAE) at the University of California, Davis campus farm to evaluate the performance of the sensor platform for measuring row crop spacing. For this test, an implement was designed to house the sensors as follows. The same IR light-beam sensor and encoder, both described in Section 2.1, were used (Figure 2). The output signals of the sensors were connected to a bidirectional digital module (NI 9403, National Instruments Co., Austin, TX, USA), while the signal encoder was connected to a digital input module (NI 9411, National Instruments Co.). Both modules were integrated into an NI cRIO-9004 (NI 9411, National Instruments Co.), and all data were recorded using LabVIEW (National Instruments Co.). In these early field trials, the team worked on three lines of a small plot of land 20 m in length, where the methodology for detecting the plants within a crop line was tested.

**Figure 3:**
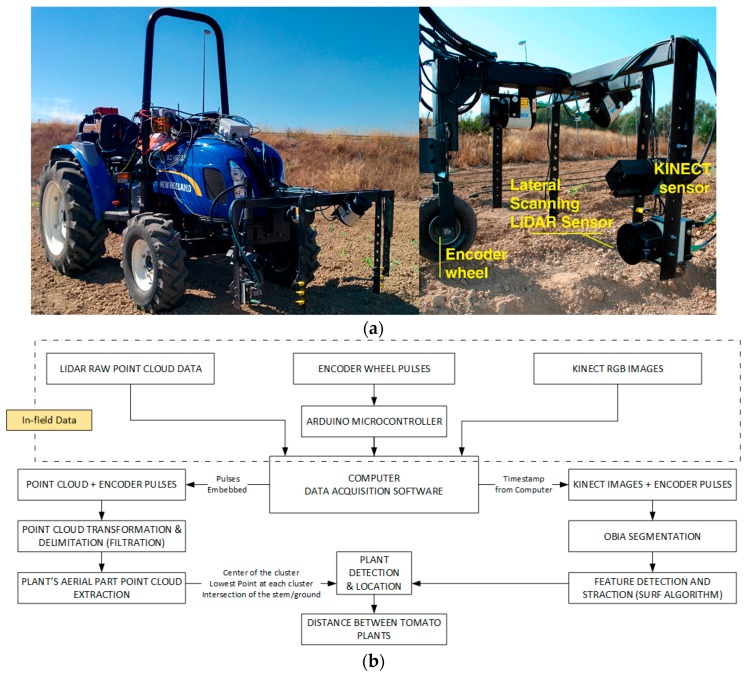
(**a**) Structure housing the sensors mounted on the tractor (left) and detail of the LiDAR and Kinect setup (right) for field trials at the University of Seville; (**b**) Progressive monitoring flowchart using LiDAR and Kinect sensors.

To continue the study of plant localization in a different scenario, additional experiments were designed at the University of Seville, in which a refinement of the LiDAR sensors and data processing were performed. These tests were conducted on several lines of tomato plants manually transplanted from trays, with the plants placed with an approximate, though intentionally non-uniform, spacing of 30 cm. Two of these lines were analysed further, one with 55 tomato plants and the other with 51, and a line of 19 wooden sticks was also placed to provide an initial calibration of the instruments. Due to the initial test conditions, where tomato plants were recently transplanted and had a height of less than 20 cm, the team built an inverted U-shaped platform attached to the front of a small tractor (Boomer 35, New Holland, New Holland, PA, USA, Figure 3). The choice of the small tractor was motivated by the width of the track, as the wheels of the tractor needed to fit on the sides of the tomato bed, leaving the row of tomatoes clear for scanning and sensors. 

As was done in the laboratory platform, the encoder described in Section 2.1 was used as an odometric system, this time interfaced with an unpowered ground wheel, to determine the instantaneous location of the data along the row. 

During the tests, the platform presented several key points that were addressed: (i) The encoder proved to be sensitive to vibrations and sudden movements, so it was integrated into the axis of rotation of an additional wheel, welded to the structure and dampened as much as possible from the vibrations generated by the tractor. In addition, the team had to reduce the slippage of the encoder wheel on the ground to avoid losing pulses; (ii) Correct orientation of the sensors was also key because the mounting angles of the LiDAR sensors would condition the subsequent analysis of the data obtained in the scans and the determination of which data contributed more valuable information; (iii) The speed of the tractor should be as low as possible remain uniform (during the test the average speed was 0.36 m/s) and maintain a steady course without steering wheel movements to follow a straight path.

### 2.4. Data-Processing Methodology

To detect precisely and determine properly the distances between plants in both laboratory and field tests, the data provided by the sensors were merged and analysed.

#### 2.4.1. Plant Characterization Using Light-Beam Sensors

The methodology followed to analyse data obtained from the light-beam curtain (which was formed by three light-beam sensors in line) was similar to that described in [21]. The algorithm outputs the moment that the beam was interrupted and associates the beam with an encoder pulse. Because the 3D plants had a wider shape at the top (leaves) than the bottom (stem), and therefore more interruptions were received, the algorithm had to be adapted to each sensor pair and each height for plant detection. To discriminate correctly between plants for the light curtain case, the developed algorithm implemented a distance range, measured in pulses from the encoder, that allowed the verification of the presence or absence of a plant after the detection of the stem, inferring that interruptions received from the sensors placed at the middle and top heights before and after the stem corresponded to the leaves and the rest of the plant structure, respectively. For the analysis of data obtained from the single pair of IR light-beam sensors, a Matlab routine (MATLAB R2015b, MathWorks, Inc., Natick, MA, USA) was developed. System calibration was performed using 11 artificial plants in the laboratory test and 122 real tomato plants in the UC Davis field test. The methodology used for the detection of tomato plants was based on the following steps:

1. Selection of Values for the Variables Used by the Programme for Detection:
  a) *Pulse_distance_relation*: This variable allowed us to convert the pulses generated by the encoder into the distances travelled by the platforms. In laboratory trials, the encoder was coupled to the shaft that provided motion to the 3D plants, and in the field, it was coupled to a wheel installed inside the structure of the platform. The conversion factors used for the tests were 1.18 and 0.98 mm per pulse for the laboratory and the field, respectively.
  b) *Detection_filter*: To eliminate possible erroneous detections, especially during field trials due to the interaction of leaves, branches and even weeds, the detections were first filtered. We filtered every detection that corresponded to an along-track distance of less than 4 mm while the sensor was active (continuous detection).
  c) *Theoretical_plant_distance*: The value for the theoretical distance between plants in a crop *line*. The value set during testing was 100 mm and 380 mm for the laboratory and the field, respectively.
  d) *Expected_plant_distance*: Expected distance between plants in a crop line was defined as the theoretical plant distance plus an error of 20%.
2. Importing of raw data recorded by the sensors (encoder and existence “1” or absence “0” of detection by the IR sensors). The conversion factor (*pulse_distance_relation*) provided the distance in mm for each encoder value.
3. Data were filtered by removing all detections whose length or distance travelled, while the sensors were active, was less than the set value (*detection_filter*). Thus, potential candidates were selected by registering the following:
  a) The distance at the start of the detection;
  b) The distance at the end of the detection;
  c) The distance travelled during detection and (iv) the mean distance during the detection, which was considered the location of the stem of the plant.
4. Distance evaluation between the current candidate and the previous potential plant:
  a) If the evaluated distance was greater than the value set (*expected_plant_distance*), we considered this candidate as a potential new plant, registering in a new matrix: the number of the plant, the detections that defined it, the midpoint location and the distance from the previous potential plant.
  b) If the evaluated distance was less than the set value (*expected_plant_distance*), plant candidate data was added to the previous potential plant, recalculating all components for this potential plant. The new midpoint was considered the detection closest to the theoretical midpoint.

#### 2.4.2. Plant Characterization Using a Side-View LiDAR

For the analysis of the data obtained from the LiDAR, it is important to mention the high complexity of its data, in both volume and format, compared with those data obtained by the light-beam. This is reflected in the following section, which explains the proposed methodology for obtaining both the aerial point clouds of the tomato rows referenced to the encoder sensor and the tomato plant identification. This is a prerequisite for tomato plant localization. For this purpose, it was necessary to pre-process the data, followed by a transformation and translation from the LiDAR sensor to the scanned point.

##### Pre-Processing of Data

(i) Data pre-processing was performed at the LiDAR sensor. 

An off-line Matlab process was used with the actual field data collected during the field experiments. Data were filtered to eliminate false positives or those that did not contribute relevant information, considering only those detections with a distance greater than 0.05 m. Later, the resulting detections were transformed from polar to Cartesian coordinates using a horizontal orientation coordinate system as a reference.

(ii) From the LiDAR sensor to the scanned point: transformations and data delimitation.

To transform the horizontal LiDAR coordinates to the actual LiDAR orientation (lateral in our case), the following steps were followed:

a) The starting points were the Cartesian coordinates obtained using the horizontal orientation as a reference $\left(x_{point}^{\prime },y_{point}^{\prime },z_{point}^{\prime }\right)$.
b) To integrate the scan from LiDAR into the platform coordinate system, a different transformation $(x_{\varphi },y_{\theta },z_{\psi })\;$was applied (see Equation (1)), considering the actual orientation of the LiDAR (see Table 3). Each LiDAR scanned point in the platform coordinate system $\left(x_{point},y_{point},z_{point}\right)$ was obtained:
  (1) $$\begin{array}{c} \left[\begin{array}{c} x_{\mathrm{point}}\\ y_{\mathrm{point}}\\ z_{\mathrm{point}}\\ 1 \end{array}\right]=\left[\begin{array}{c} x_{\mathrm{point}}^{\prime }\\ y_{\mathrm{point}}^{\prime }\\ z_{\mathrm{point}}^{\prime }\\ 1 \end{array}\right]\times \left[\begin{array}{cccc} 1 & 0 & 0 & 0\\ 0 & \cos(x_{\varphi }) & \sin(x_{\varphi }) & 0\\ 0 & -\sin(x_{\varphi }) & \cos(x_{\varphi }) & 0\\ 0 & 0 & 0 & 1 \end{array}\right]\times \left[\begin{array}{cccc} \cos(y_{\theta }) & 0 & -\sin(y_{\theta }) & 0\\ 0 & 1 & 0 & 0\\ \sin(y_{\theta }) & 0 & \cos(y_{\theta }) & 0\\ 0 & 0 & 0 & 1 \end{array}\right]\times \\ \left[\begin{array}{cccc} \cos(z_{\psi }) & \sin(z_{\psi }) & 0 & 0\\ -\sin(z_{\psi }) & \cos(z_{\psi }) & 0 & 0\\ 0 & 0 & 1 & 0\\ 0 & 0 & 0 & 1 \end{array}\right]\times \left[\begin{array}{cccc} 1 & 0 & 0 & 0\\ 0 & 1 & 0 & 0\\ 0 & 0 & 1 & 0\\ Enc_{t} & 0 & 0 & 1 \end{array}\right] \end{array}$$

Once transformed, the x translation was applied to coordinates obtained for the actual LiDAR orientation. The encoder values recorded at each scan time were used to update the point cloud x coordinate related to the tractor advance. Additionally, the height values (*z* coordinate) were readjusted by subtracting the minimum obtained.

##### Plant Localization

The 3D point cloud processing was performed at each stick or tomato row. Thus, using manual distance delimitation, point clouds were limited to the data above the three seedbeds used during the tests.

(i) Aerial Point Cloud Extraction

The aerial point data cloud was extracted using a succession of pre-filters. First, all points that did not provide new information were removed using a gridding filter, reducing the size of the point cloud. A fit plane function was then applied to distinguish the aerial points from the ground points. In detail, the applied pre-filters were:

a) Gridding filter: Returns a downsampled point cloud using a box grid filter. GridStep specifies the size of a 3D box. Points within the same box are merged to a single point in the output (see Table 4).
b) pcfitplane [23]: This Matlab function fits a plane to a point cloud using the M-estimator SAmple Consensus (MSAC) algorithm. The MSAC algorithm is a variant of the RANdom SAmple Consensus (RANSAC) algorithm. The function inputs were: the distance threshold value between a data point and a defined plane to determine whether a point is an inlier, the reference orientation constraint and the maximum absolute angular distance. To perform plane detection or soil detection and removal, the evaluations were conducted at every defined evaluation interval (see Table 4).

Table 4 shows the parameter values chosen during the aerial point cloud extraction. The chosen values were selected by trial and error, selecting those that yielded better results without losing much useful information.

(ii) Plant Identification and Localization

• Plant Clustering

A k-means clustering [24] was performed on the resulting aerial points to partition the point cloud data into individual plant point cloud data. The parameters used to perform the k-means clustering were as follows:

○ An initial number of clusters: Floor ((distance_travelled_mm/distance_between_plants_theoretical) + 1) × 2
○ The squared Euclidean distance for the centroid cluster. Each centroid is the mean of points in the cluster.
○ The squared Euclidean distance measure and the k-means++ algorithm were used for cluster centre initialization.
○ The clustering was repeated five times using the initial cluster centroid positions from the previous iteration.
○ Method for choosing initial cluster centroid positions: Select k seeds by implementing the k-means++ algorithm for cluster centre initialization.

A reduction in the number of clusters was determined directly by evaluating the cluster centroids. If pair of centroids were closer than the *min_distance_between_plants*, the process was repeated by reducing the number of clusters by one (Table 5).

A clustering size evaluation was performed, excluding clusters smaller than *min_cluster_size*.

(iii) Plant Location

Three different plant locations were considered:

- Centre of the cluster
- Location of the lowest point at each plant cluster
- Intersection of the estimated stem line and ground line
  ○ By dividing the aerial plant data in slices defined by “histogram jumps”, the x limits on the maximal number of counts were obtained. For the z limits, data belonging to the bottom half of the aerial point cloud were considered.
  ○ The remaining data inside these limits were used to obtain a line of best fit, which was considered the stem line.
  ○ Plant location was defined as the intersection between the stem line and the corresponding ground line obtained previously from the MSAC function.

#### 2.4.3. Validation of Plant Location Using RGB Kinect Images

To obtain the distance between the stems of two consecutive plants using the Kinect camera, it is necessary to characterize the plants correctly and then locate the stems with RGB images from the Kinect camera. This characterization and location of the stem was conducted as follows: a sequence of images of the entire path was obtained (~250 images in each repetition), where the camera’s shooting frequency was established steadily in at 1-s intervals. Obtaining the string with the timestamp of each image was a key aspect of the routine developed in Matlab, as this string would later be used for integration with the LiDAR measurement. The relationship between the timestamp and its corresponding encoder value was used to spatially locate each image (*x*-axis, corresponding to the tractor advance). 

**Figure 4:**
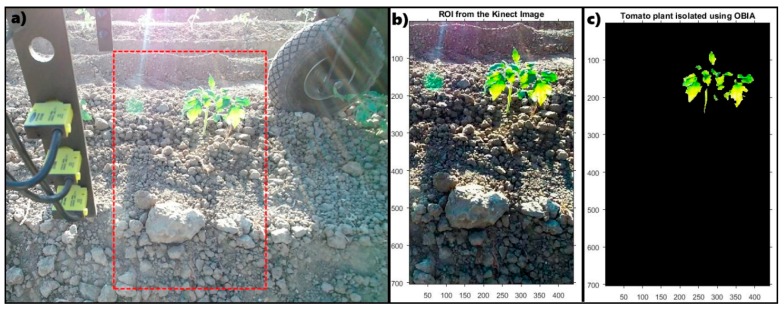
(**a**) raw image captured by the Kinect sensor with the ROI indicated; (**b**) ROI with white balance and saturation adjustment; (**c**) OBIA resulting image with isolated plant pixels.

Image processing techniques applied in the characterization of tomato plants from Kinect images generally followed these steps:

(i) According to [25], the first step in most works regarding image analysis is the pre-processing of the image. In this work, an automatic cropping of the original images was performed (Figure 4a), defining a ROI. Because the test was conducted under unstructured light conditions, the white balance and the saturation of the cropped image (Figure 4b) were modified;
(ii) Next, the image segmentation step of an object-based image analysis (OBIA) was performed to generate boundaries around pixel groups based on their colour. In this analysis, only the green channel was evaluated to retain most of the pixels that define the plant, generating a mask that isolates them (Figure 4c) and classifying the pixels as plant or soil pixels. In addition, morphological image processing (erosion and rebuild actions) was performed to eliminate green pixels that were not part of the plant;
(iii) Each pair of consecutive images was processed by a routine developed in Matlab using the Computer Vision System Toolbox. This routine performs feature detection and extraction based on the Speeded-Up Robust Features “SURF” algorithm [26] to generate the key points and obtain the pairings between characteristics of the images.

Once the plant was identified in each image, the location of the stem of each plant was defined according to the following subroutine:

(1) Calculating the distance in pixels between two consecutive images, as well as the encoder distance between these two images, the “forward speed” in mm/pixel was obtained for each image;
(2) For each image, and considering that the value of the encoder corresponds to the centre of the image, the distance in pixels from the stem to the centre of the image was calculated. Considering whether it was to the left or to the right of the centre, this distance was designated positive or negative, respectively;
(3) The stem location was calculated for each image using the relation shown in Equation (2) below;
(4) Plant location was obtained for each image in which the plant appeared; the average value of these locations was used to calculate the distance between plants:
  (2) $$\mathrm{Stem}\;\mathrm{Location}\;=\;\mathrm{Encoder}\;\mathrm{Value}\;\pm \;\mathrm{Distance}\;(\mathrm{from}\;\mathrm{stem}\;\mathrm{to}\;\mathrm{centre})\;\times \;\mathrm{motion}\;\mathrm{relation}\left(\frac{\mathrm{mm}}{\mathrm{pixel}}\right)$$

## 3. Results

### 3.1. Laboratory Test Results

**Figure 5:**
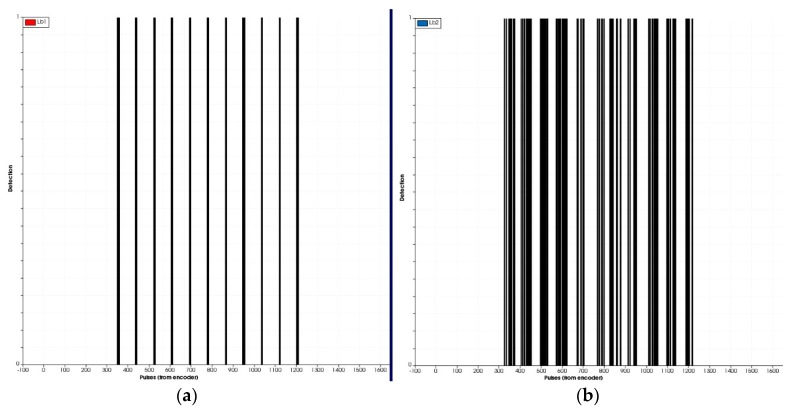
(**a**) Stem detection of the 11 artificial plants through the lower pair of light-beam sensors. (**b**) Detection of the aerial part (leaves and branches) of the 11 artificial plants through the centre pair of light-beam sensors.

Several laboratory tests were conducted to properly adjust and test the sensors. As an example, Figure 5 shows various profiles representing detections, comparing the pair of light-beam sensors placed on the bottom (stem detection) with those placed in the middle, detecting the aerial part of the 3D plant. Detections of the third pair of sensors, placed above the plants, were not considered relevant because most of their beam was above the plants. Figure 5 also shows that the detection of the stem using a single pair of light-beam located at a lower height with respect to the soil was more effective and robust than trying distinguish between different plants based on the detection of the aerial part of the plant. From our point of view and in agreement with the results in [21], this justifies the use of a single pair of light-beam sensors for the field tests rather than the use of the curtain mode (3 pairs as originally tested in the laboratory). The algorithm to analyze data from a single light-beam is also simpler and faster, being more adequate for real-time usage.

**Figure 6:**
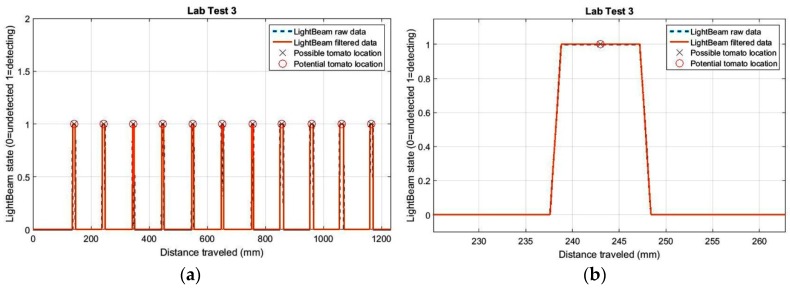
(**a**) Positions of the stems of the 11 plants in the laboratory test; (**b**) Average plant position for a distance of 11 mm.

Figure 6 shows the positions of the stem detections and the estimated distances between them. In all laboratory tests, 100% of the stems were detected using the light-beam sensor. Notably, under laboratory conditions, there were no obstacles in the simulated crop line, which does not accurately represent field conditions. Figure 6b shows the stem diameter measured from a test, with an estimated average diameter of 11.2 mm for an actual value of 10 mm.

**Figure 7:**
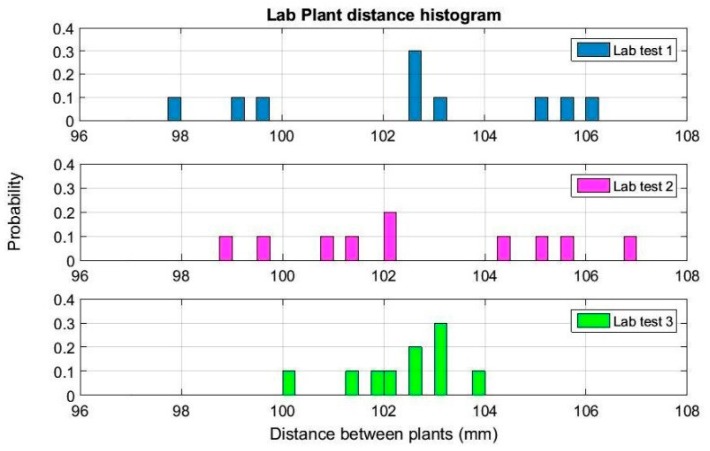
Histogram of measured distances between 3d plant stems during the adjusting process of sensors on the detection platform in the laboratory.

Three laboratory tests corresponding to three different stages of the light-beam sensor adjustment process are shown in Figure 7. 

The histogram in this figure represents the estimated distances between plant stems, where the average distance is 102.5 mm when the real distance between plants was 100.0 mm. The average standard deviation for the three trials was 2.2 mm. For test 3 (last histogram in Figure 7), all distance values between plants were estimated between 100.1 mm and 103.8 mm.

**Figure 8:**
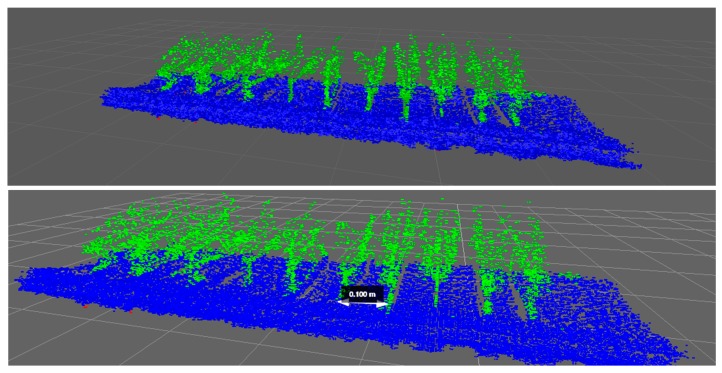
Point cloud representation of artificial 3D plants obtained using a lateral-scanning LiDAR sensor during laboratory tests.

For the results obtained by the LiDAR in the laboratory, as described in Section 2.2, multiple scanning configurations and orientations were tested. After a visual comparison of the results of these tests, lateral-scanning orientation gave the best point clouds, and its plant representation and spacing measurements were therefore more accurate. Figure 8 shows the point cloud representation of one of the laboratory scans made using the LiDAR sensor with lateral orientation.

### 3.2. Field Tests Results

**Figure 9:**
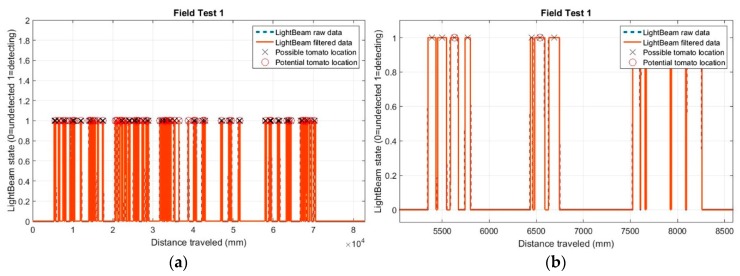
(**a**) Detections and positions of the 41 tomato plants in first field test with IR sensors; and (**b**) detail of the positions of potential plants.

Preliminary tests results using the light-beam sensors are presented in Figure 9a. Unlike laboratory detections, in the field data, the determined distances between stems were more variable. This variability is mainly due to crop plants missing from the line or the presence of a weed very close to the stem of the detected plant. Figure 9b reveals that several positions for potential plants (marked with a cross) were established, while only one plant was present (marked with a circle).

During the first field test, 41 detections occurred when there were 32 real plants growing in the line; thus, for this trial, the error in the number of detections was 22% (Table 6). We speculate three possible causes of this error in the initial test: the plants were very close together, soil clods were detected between plants, or the evaluation of the distance between plants for the selection of potential plants was not optimal. However, in trial two, 32 stem detections occurred when 34 real plants were in the line, resulting in a 94% success rate. For test three, the accuracy was 98%.

Regarding the experiments conducted to estimate the response of the LiDAR system in real field conditions, the detection system was used on three lines (one with wooden sticks and two with tomatoes). The system was intended to detect the presence of plant stems correctly and to distinguish between the foliage and the stem. In addition, this system was used to measure the distances between plants accurately, providing valuable information for future herbicide treatments. 

Because the use of the laser scanner for detection implies the generation of a very dense cloud of points, in which not all data are relevant, an initial filtering was performed as explained above. Table 7 presents the number of original points obtained by eliminating the seedbed, and those considered to be representative of the aerial part of the plant, which comprised only 4.5% of the total.

Regarding plant detection, Table 8 summarizes the results of some of the tests, implementing the methodology explained previously. Based on these data, it is observed that 100% of the sticks have been correctly detected, without obtaining false positives or negatives. When this analysis was performed on tomato plants, about 3–21% of false positive or negative detections were found. A plant detected by the LiDAR was defined as a false positive when the estimated plant centre was more than half of the plant spacing from the real plant centre. 

**Figure 10:**
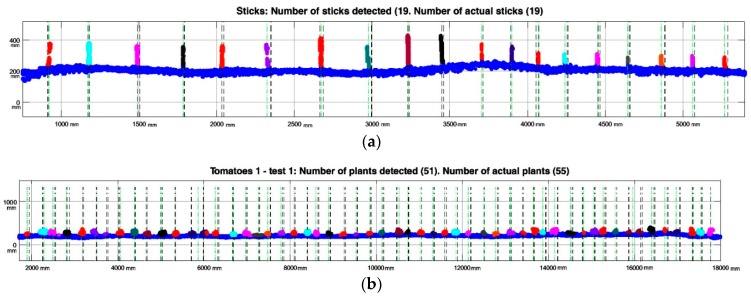
Detection results on three tested lines. Green dotted lines represent the cluster centre and black dotted lines the real plant interval obtained by the Kinect image (location ± Std.). (**a**) 19 stick detections using LiDAR; (**b**) 51 plants detected during Test 1 in row 1.

Related to the data presented in Table 8, Figure 10 below shows stick and plant LiDAR detection results in three rows. Platform advance information (*x*-axis on the plot) provided by the encoder is given in mm. Each plant/stick detected is marked with different colour (note the overlapping on some plants, explaining false negative and false positive detections). 

**Figure 11:**
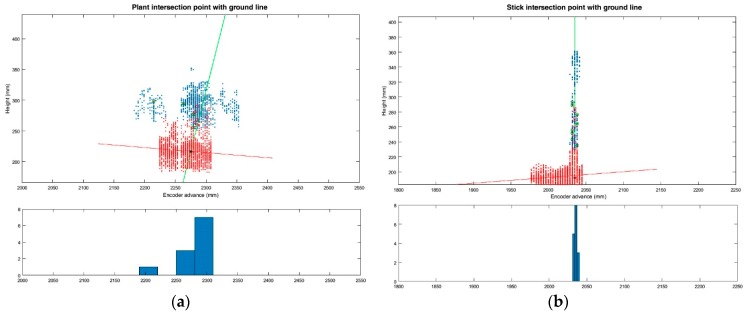
Plant location method based on the intersection of stem line and ground line. (**a**) Intersection point between the average aerial part of the plant line (green line) and the average ground line (red line); (**b**) Intersection point between the stick aerial part line (green line) and the ground line (red line). Both histograms of aerial part of the plant points are shown on the bottom.

As explained in Section 2.4.2.2, the plant location method based on the point-to-ground intersection has been evaluated (in addition to the lowest-point and centre-of-cluster methods). Figure 11 shows an example of the insertion point obtained from the intersection of the two lines (aerial part and soil line) in tomato plants and sticks.

Plant locations obtained from the LiDAR and Kinect data are shown in Table 9. As explained in Section 2.4.3, when processing the Kinect images, a value of the encoder was automatically selected for each image. Mean values are obtained from the difference between the actual location and the location obtained with the LiDAR. The negative mean value obtained for Tomatoes 2 with filter Test 2 for the intersection of the stem and ground line method means that the LiDAR detected the plant at a distance greater than the actual distance (obtained from the Kinect). High standard deviation values can be explained due to the high variability found in the encoder values assigned to each plant or stick during the image processing.

**Figure 12:**
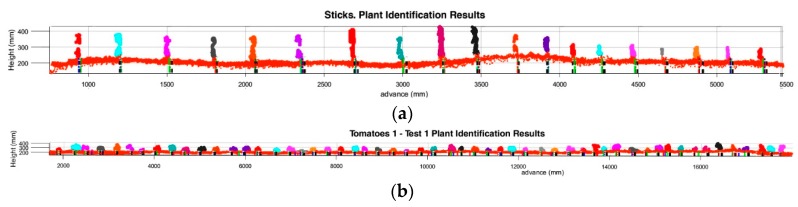

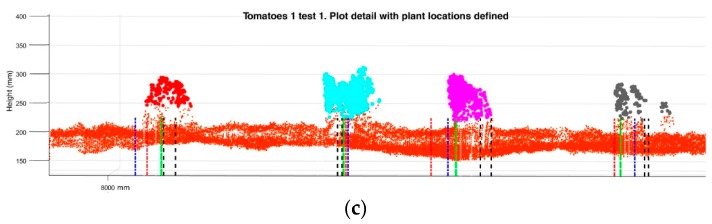
Plant and sticks location results obtained by the three different methods: Centre of the cluster; lowest point; and ground-plant intersection. (**a**) Sticks row data using LiDAR; (**b**) Tomato plants detected during Test 1 on row 1; (**c**) Detail of the aerial point cloud of tomato plants generated by the LiDAR during Test 1 on row 1. Each plant location method is marked with different coloured dotted line.

Figure 12 shows the plant identifications in each line during the different tests. For each detection previously shown in Figure 10, tags for each of the localization methods are shown by coloured lines. 

The centre of the cluster is marked in green, the lowest point in blue, and the intersection in red. Encoder stick/plant locations by the Kinect image (location ± Std.) are both marked in black lines, and their distances are represented by black doted.

## 4. Conclusions

A combination of optical sensors mounted on a frame on a tractor, which are capable of detecting and locating plants and utilizing ground-wheel odometry to determine a local reference system, was successfully developed and tested. Combining the use of affordable sensors (light-beam and Kinect) with more expensive ones (LiDAR), a laboratory setup platform and two field test platforms have been created. The following conclusions were drawn based upon the results of this research:

- The results obtained from the precise locations of the plants allow us to consider the viability of further methods capable of performing individualized treatments for each of the plants, or accurate agrochemical treatments to remove existing weeds between crop plants in the same row.
- A light-beam detection system was improved by reduction of the number of sensor pairs used. The reduction from three pairs to one pair had no effect on the desired plant detection results. In the field tests, 98% precision of detection was obtained, similar to that obtained in [21], showing that this is a robust technology and can be deployed in the field. Also, light-beam detection data allows faster processing than the LiDAR, so it could be used in real-time applications.
- Based on the methodology presented in the analysis of the data from the LiDAR and the Kinect sensors, different considerations can be established regarding the location of the same plant. According to the structure and morphology of the plant, it is assumed that the aerial part will not always be vertically in line with the stem (in fact, in the tests done this occurred frequently). For this reason, one of three proposed locations can be established as the location of the plant: the aerial part, the cluster centroid of the filtered point cloud or the insertion of the stem. Depending on the type of treatment to be made, one of these locations could be more interesting to evaluate than the others. For example, if a variable foliar herbicide treatment is to be applied (discriminating between weeds and crop plants), the distance between the aerial parts of the plants should be given greater weight in the application system (to avoid applications on the crop plants and maximize efficiency by ensuring the herbicide is applied to weeds). In the case of physical weed-removal systems, as proposed in [8], priority should be given to the location of stem insertion, and adjustments should be made to detect this element more precisely.
- The high volume of data generated by the more accurate sensors, such as the LiDAR used in this work, can be a hurdle for automatic weed detection machines when working in real time. However, it is important to emphasize the exponential growth of the processing algorithms available for the researcher, which can significantly reduce the time required for point cloud data analysis. Reducing the density of information necessary while continuing to give accurate information is an interesting subject for future work.
- The precise locations of the plants were determined using an encoder. The use of this type of sensor is vital to implementation of low-cost referencing. Correct localization and integration to enable determination of reliable reference of the movement is of great importance.
- Agricultural use of affordable electronic components will lower costs in the near future, but the authors conclude that these systems must be robust and provide a rapid response and processing times.

## Figures and Tables

**Table 1:** IR light-beam sensor features.

Operational Voltage (V)	10–30 V
Detection range (m)	30 m
Response time (milliseconds)	1 ms
Sinking and sourcing outputs (mA)	150 mA

**Table 2:** LMS 111 technical data.

Operational Range	From 0.5 to 20 m
Scanning field of view	270°
Scanning Frequency	50 Hz
Angular resolution	0.5°
Light source	905 nm
Enclosure rating	IP 67

**Table 3:** Transformation and translation values applied to LiDAR data with a lateral orientation.

Roll “φ” (°)	Pitch “θ” (°)	Yaw “ψ” (°)	x Translation (m)
0	−180	0	Enct

**Table 4:** Aerial point cloud extraction parameters selected.

Test	Grid Step (m^3^)	MSAC				
		Theoretical Distance Between Plants (m)	Evaluation Intervals (m)	Threshold (m)	Reference Vector	Maximum Absolute Angular Distance	
Sticks	(3 × 3 × 3) × 10^−9^	0.240	0.08	0.04	[0,0,1]	5	
Tomatoes 1		0.290	0.096				
Tomatoes 2							

**Table 5:** Plant identification and stem identification parameters.

Minimum Distance between Plants	Minimum Cluster Size	Histogram Jumps (mm)
Distance_between_plants_theoretical×0.2	5	4

**Table 6:** Plant detection ratio in field trials.

Test	Real Plants	Detected Plants	% Accuracy
First test	32	41	78
Second test	34	32	94
Third test	48	49	98

**Table 7:** Point cloud reduction during plant point cloud extraction.

Test	Seedbed Delimit	Gridding	Plant Points
Stick: Test 1	44,708 (100%)	40,903 (91.5%)	1667 (3.7%)
Tomatoes 1: Test 1	374,963 (100%)	328,593 (87.6%)	14,720 (3.9%)
Tomatoes 1: Test 2	237,396 (100%)	220,714 (93%)	13,098 (5.5%)
Tomatoes 2: Test 2	3,807,858 (100%)	340,051 (89.3%)	18,706 (4.9%)

**Table 8:** Plant and sticks detection results.

Test	Plant Location Method	Correctly Detected	False Positive	False Negative
Stick with filter Test 1	Centre of the cluster	19	0	0
	Lowest point	19	0	0
	Intersection of stem line and ground line	19	0	0
Tomatoes 1 with filter Test 1	Centre of the cluster	49	2	6
	Lowest point	48	3	7
	Intersection of stem line and ground line	46	5	9
Tomatoes 1 with filter Test 2	Centre of the cluster	53	6	2
	Lowest point	52	7	3
	Intersection of stem line and ground line	47	12	8
Tomatoes 2 with filter Test 2	Centre of the cluster	51	5	0
	Lowest point	48	8	3
	Intersection of stem line and ground line	42	14	9

**Table 9:** Mean and standard deviation (Std.) of the plant and stick locations during the tests.

Test	Plant Location Method	Mean (mm)	Std. (mm)
Stick—Test 1	Centre of the cluster	8.32	10.09
	Lowest point	7.25	8.47
	Intersection of stem line and ground line	6.15	9.45
Tomatoes 1—Test 1	Centre of the cluster	20.74	40.37
	Lowest point	10.06	51.72
	Intersection of stem line and ground line	5.34	62.65
Tomatoes—Test 2	Centre of the cluster	30.42	37.03
	Lowest point	35.48	52.90
	Intersection of stem line and ground line	27.60	50.36
Tomatoes 2—Test 2	Centre of the cluster	1.33	41.49
	Lowest point	10.00	61.86
	Intersection of stem line and ground line	−5.95	53.32
